# Beyond survival: Redefining successful aging in the era of medical complexity

**DOI:** 10.1371/journal.pbio.3003784

**Published:** 2026-05-14

**Authors:** Karin Modig, Marcus Ebeling

**Affiliations:** 1 Unit of Epidemiology, Institute of Environmental Medicine, Karolinska Institute, Stockholm, Sweden; 2 Medical Demography, Max Planck Institute for Demographic Research, Rostock, Germany; Harvard University T H Chan School of Public Health, UNITED STATES OF AMERICA

## Abstract

Modern medicine has transformed not only how long we live, but also how we age, with more people surviving to old age with chronic disease. This Perspective examines how aging, health, and care should be redefined to reflect these increasingly complex later lives.

## Teaser

A common misconception is that increasing longevity reflects slower aging. Instead, most longevity gain comes from medical advances that allow survival with disease, rather than changes to the biology of aging itself, challenging how we study aging and health.

Modern medicine has not only extended life, it has also reshaped the very nature of aging. Over the past century, life expectancy has increased substantially across much of the world, driven by reductions in infant and infectious disease mortality and by sustained improvements in the prevention and treatment of chronic diseases in later life. In high-income countries today, nearly everyone survives into old age, and individual life spans have never been so predictable. Yet, by extending survival, modern medicine has also transformed the biological and clinical reality of aging.

Survival has improved across almost every major disease category, and conditions that once killed swiftly are now managed for years, often decades. Notably, improvements seem to primarily concern the final manifestations of underlying disease. For example, the incidences of myocardial infarction and stroke have declined substantially, whereas the incidences of hypertension, hypercholesterolemia, heart failure, and atrial fibrillation have not decreased at the same pace [1–3]. Nor have the incidences of dementia or osteoporosis, although modest improvements have taken place [4,5]. The key question is therefore: do these developments reflect true modification of the biological aging process itself, as conceptualized in the hallmarks of aging [6].

**Fig 1:**
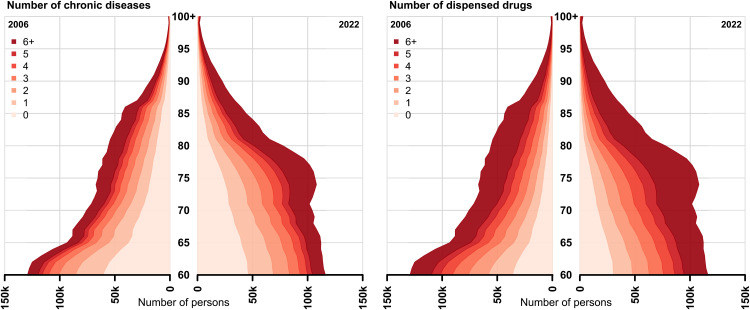
Total population size by number of chronic diseases and number of dispensed drugs in Sweden in 2006 and 2022. The number of chronic diseases (left) was calculated based on inpatient and specialist outpatient diagnoses over a 5 year period (2006: 2001-12-31–2006-12-31; 2022: 2017-12-31–2022-12-31) with register data covering the total Swedish population. An adjusted version of the chronic condition indicator from the Health Cost & Utilization Project (HCUP) was used to identify chronic diseases. The number of dispensed drugs (right) was counted as the number of unique 3-level ATC codes observed over a 6 months period (2006: 2006-07-01–2006-12-31; 2022: 2022-07-01–2022-12-31) in the Swedish medical prescription register.

The success of treating diseases and saving lives has quietly transformed aging itself, making older populations today biologically and clinically different from those of 50 years ago [7,8]. Medical progress has not only extended life, it has also altered who survives into old age. Figure 1 illustrates that the population aged between 60 and 100 years in Sweden has grown larger between 2006 and 2022 (population aging), but that the growth is concentrated in the red-shaded areas, representing disease burden as measured through diagnoses or medications.

To avoid an overly negative narrative, it is important to recognize that although multimorbidity has increased, the burden associated with these conditions is likely lower for many individuals than in previous generations. For example, recent OECD data show that a majority of older adults with chronic conditions report good or very good social functioning, and high-income countries have observed improvements or stability in activity limitations over recent years [9]. These trends likely reflect improved treatments and disease management strategies that help older adults maintain function and perceived health, despite rising disease prevalence. Beyond improved survival, this may represent a second major achievement of modern medicine: a growing disconnection between the presence of disease and its functional consequences. Thus, the continuum between health and illness has widened, making the traditional binary perspective on health—healthy versus unhealthy—too simplistic.

Much biomedical research still conceptualizes aging as a process of biological decline, often modeled through single-disease or pathway-specific frameworks. There is a risk that precision medicine (seeking to optimize treatment based on individual biological profiles) may intensify disease-specific care without adequately integrating whole-person complexity. Our scientific models of aging have not yet fully adapted to the demographic and clinical realities created by extended survival with chronic disease. Playing devil’s advocate, one could argue that the implicit goal of medicine in the later part of life has gradually shifted from curing acute illness to postponing, and eventually even prolonging, death.

Multimorbidity and polypharmacy may also contribute to what could be described as iatrogenic aging: aging shaped not only by underlying biological processes but also by the cumulative effects of medical intervention. Modern medical interventions interact with each other and with underlying conditions, potentially altering physiological trajectories in ways that remain only partly understood. While each intervention is typically evidence-based and beneficial when considered in isolation, their combined effects across decades of life and across multiple conditions have rarely been studied systematically. As a result, the aging bodies we observe today may reflect not only the biology of aging itself but also the biological imprint of sustained medical care. This perspective does not diminish the enormous benefits of modern medicine. Rather, it highlights that medicine has become an active participant in shaping the biological and experiential landscape of old age. Understanding these interactions may therefore become an increasingly important frontier in aging research.

Another question is whether ethical and social frameworks are evolving at the same pace as the therapeutic advances that treat and prolong life with chronic diseases. As survival increases under conditions of medical complexity, we need to reconsider what constitutes successful aging—biologically, clinically, and socially, particularly given the lack of clear and consistent definitions of health and healthspan [10]. From a scientific perspective, we rarely ask whether prolonged survival under a high treatment burden aligns with patients’ values and priorities, even though this question is becoming increasingly central in general practice and geriatric medicine. Policy and research agendas may therefore need to shift from maximizing life span towards optimizing the quality and meaning of the years lived under conditions of medical complexity [11]. But who defines what constitutes “optimized” life years? Ensuring that older adults themselves meaningfully shape research priorities and clinical goals is more important than ever. In some cases, this may require moving beyond a singular focus on prolongation towards a more explicit engagement with patients’ preferences regarding the limits of treatment and the conditions under which life should be allowed to end. Moreover, we may need to reconsider the metrics used to study health and disease. If diagnoses no longer mean the same thing as they once did in terms of experienced disease burden, and if an additional year of survival is associated with a different quality of life than in previous generations, then the outcomes we traditionally measure may need to evolve as well. It is not enough to develop frameworks and guidelines that address the whole-person living with multiple interacting conditions, we must also develop outcome measures capable of capturing this complexity.

At the same time, navigating health in later life will likely become increasingly complex as medical options, treatment strategies, and clinical knowledge continue to expand. Achieving good outcomes will require a careful balance between individual responsibility and system responsibility. Without such balance, individuals with higher education, stronger social networks, or access to informal caregivers may benefit disproportionately compared with those who lack these supporting structures. Without cautious attention to these dynamics, the expanding possibilities of medicine may inadvertently widen inequalities in both longevity and quality of life in old age.

The result of these developments is a fundamentally new landscape of aging. Increasing numbers of individuals now reach advanced ages while living with multiple chronic conditions and long-term treatments. Yet many of the conceptual frameworks used to study aging were developed for a world in which far fewer people survived to such ages, and with less complex disease/medical histories. As longevity continues to increase, a central challenge for aging research will therefore be to redefine what constitutes success in these added years, and to develop frameworks, metrics, and care models that better reflect the biological and lived realities of extreme longevity.
